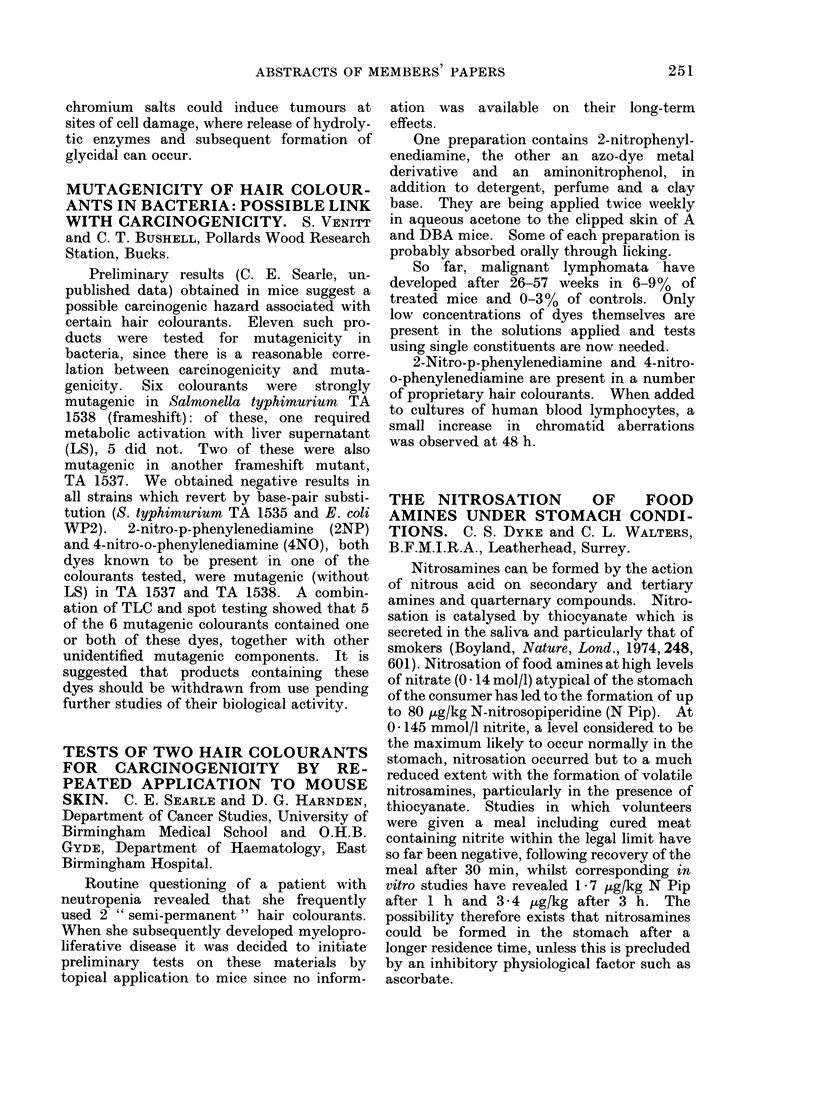# Proceedings: Tests of two hair colourants for carcinogenicity by repeated application to mouse skin.

**DOI:** 10.1038/bjc.1975.192

**Published:** 1975-08

**Authors:** C. E. Searle, D. G. Harnden, O. H. Gyde


### TESTS OF TWO HAIR COLOURANTS FOR CARCINOGENICITY BY REPEATED APPLICATION TO MOUSE SKIN.

C. E. SEARLE and D. G. HARNDEN, Department of Cancer Studies, University of Birmingham Medical School and O.H.B. GYDE, Department of Haematology, East Birmingham Hospital.

Routine questioning of a patient with neutropenia revealed that she frequently used 2 " semi-permanent " hair colourants. When she subsequently developed myeloproliferative disease it was decided to initiate preliminary tests on these materials by topical application to mice since no information was available on their long-term effects.

One preparation contains 2-nitrophenylenediamine, the other an azo-dye metal derivative and an aminonitrophenol, in addition to detergent, perfume and a clay base. They are being applied twice weekly in aqueous acetone to the clipped skin of A and DBA mice. Some of each preparation is probably absorbed orally through licking.

So far, malignant lymphomata have developed after 26–57 weeks in 6–9% of treated mice and 0–3% of controls. Only low concentrations of dyes themselves are present in the solutions applied and tests using single constituents are now needed.

2-Nitro-p-phenylenediamine and 4-nitro-o-phenylenediamine are present in a number of proprietary hair colourants. When added to cultures of human blood lymphocytes, a small increase in chromatid aberrations was observed at 48 h.